# Editorial: Novel drug-designing approaches to combat antimicrobial resistance

**DOI:** 10.3389/fmolb.2023.1342702

**Published:** 2024-01-11

**Authors:** Ghazala Muteeb, Md Tabish Rehman, Bibhusita Pani, Rizwan Hasan Khan

**Affiliations:** ^1^ Department of Nursing, College of Applied Medical Science, King Faisal University, Al-Ahsa, Saudi Arabia; ^2^ Department of Pharmacognosy, College of Pharmacy, King Saud University, Riyadh, Saudi Arabia; ^3^ Grossman School of Medicine, New York University, New York, United States; ^4^ Interdisciplinary Biotechnology Unit, Aligarh Muslim University, Aligarh, India

**Keywords:** immunoinformatics, antimicrobial resistance, *in-silico*, African catfish antimicrobial peptides (ACAPs), structure-activity relationship (SAR)

## Introduction

The global emergence of resistance in microbes in hospitals, as well as community settings, poses a serious threat to the healthcare system. Antimicrobial resistance (AMR) is defined as the ability of a microorganism to resist the adverse effects of a drug against which it was earlier susceptible. The non-judicious use and over-the-counter sale of antibiotics have led to the development of resistance in bacteria, employing various mechanisms such as the expression of beta-lactamases, drug-efflux pumps, alteration of drug-targeted pathways, biofilm formation, and quorum sensing. The scarcity of new antimicrobials in the market further exacerbates the problem. The traditional approach of developing new antimicrobials is time-consuming and non-economical. Therefore, the repurposing of already available drugs/inhibitors for other diseases may serve as a viable approach to identify novel treatment options for infections caused by resistant microbes.

Antimicrobial resistance, by itself, is not a new phenomenon; it is an evolutionary process. The challenge is to develop new antimicrobials and employ new approaches to keep pace with ever-increasing AMR, or else we risk returning to the pre-antibiotic era. In silico approaches to identify novel molecules against AMR may expedite the drug discovery and design process. Structural bioinformatics, high-throughput virtual screening, molecular docking, and molecular dynamics simulations may help identify potential drug molecules. The structure-activity relationship (SAR) may further guide the development of more potent drug molecules with minimal side effects. Researchers have explored new ideas and strategies to advance the identification of active biomolecules that may act either alone or in combination with conventional antibiotics. Progressive research on the design and development of novel antimicrobials is crucial to address the urgent need in the ongoing battle against AMR. In the relentless battle against antimicrobial resistance (AMR), a rapidly evolving adversary, the imperative for innovative drug-designing approaches has never been more critical. Traditional antibiotics are increasingly losing efficacy, leading to a global health crisis where once-treatable infections become formidable threats. This urgency has spurred groundbreaking research, ushering in novel strategies and cutting-edge technologies to design drugs capable of overcoming the resilience of antimicrobial-resistant pathogens.

In the realm of combating bacterial persistence, Civera and Sattin research delves into the sophisticated strategies employed by bacteria to withstand harsh conditions and antibiotic treatments. The study focuses on the bet-hedging strategy known as persistency, emphasizing its potential role in chronic infections and the evolution of resistant bacterial strains. By spotlighting the regulatory role of the RSH superfamily enzymes, particularly RelSeq from *Streptococcus equisimilis*, the researchers lay the groundwork for understanding the molecular mechanisms driving persisters. The exploration of the synthetase-ON structure of RelSeq unveils a partially active state, paving the way for subsequent steps in virtual screening and ligand design studies. This multifaceted approach not only sheds light on bacterial persistence but also opens avenues for the discovery of innovative antimicrobial compounds.

Amidst the escalating crisis of antimicrobial resistance, the study on African catfish antimicrobial peptides (ACAPs) stands out as a beacon of hope (Okella et al.). Recognizing the potential of antimicrobial peptides (AMPs) as a viable alternative to traditional antibiotics, the study evaluates the pharmacokinetic profiles of ACAPs using ADMET scores. This careful evaluation identifies two promising peptides, ACAP-IV and ACAP-V, with favorable profiles. The subsequent docking studies against bacterial protein targets provide insights into their potential mode of action. The synthesis and *in vitro* validation of these peptides underscore their antibacterial activity, with ACAP-IV exhibiting heightened efficacy against *Escherichia coli* and *Staphylococcus aureus*. This holistic approach, blending computational screening with experimental validation, not only highlights potential leads for various industries but also showcases the power of interdisciplinary strategies in the pursuit of effective antimicrobial agents.

Vaccine development stands as a critical frontier in the global effort to overcome antibiotic resistance. Unlike traditional antibiotics that target specific bacterial structures, vaccines stimulate the body’s immune system to recognize and neutralize pathogens, preventing infection. The study by Gouda et al. addressing *Klebsiella pneumoniae* and *Pseudomonas aeruginosa* unfolds a multifaceted approach towards vaccine development. Recognizing the lack of effective vaccines against these pathogens, the researchers strategically select potential epitope candidates. The study addresses physicochemical features, antigenicity, toxicity, allergenicity, and solubility, crucial aspects for potential human use. Computational analyses, molecular docking studies, and subsequent validation through molecular dynamics simulations reveal the designed multitope vaccine’s potential. The comprehensive approach, spanning computational design to experimental validation, lays the groundwork for future advancements in vaccine development against challenging bacterial pathogens. Meanwhile, Ashgar et al. integrate immunoinformatics with a subtractive proteomics approach against *Streptococcus pneumoniae*. Their study identifies antigenic proteins and designs a multi-epitope vaccine targeting penicillin-binding protein and ATP synthase subunit. The inclusion of epitopes results in a comprehensive vaccine. To enhance immune responses, the vaccine is coupled with a cholera enterotoxin subunit B adjuvant. The vaccine’s stability, assessed through molecular docking and molecular dynamic simulations, highlights its potential efficacy. The comprehensive *in silico* analysis lays the groundwork for future experimental validations, providing a promising avenue for the development of a multi-epitope *S. pneumoniae* vaccine. Further, an immunoinformatics and reverse vaccinology approach was adopted by Choudhury et al. to develop a vaccine against monkeypox. The study navigates through the interactions of viral proteins with TLR2 and TLR4 to identify highly immunogenic proteins. Highly immunogenic proteins are selected for epitope mapping, and a multi-epitope vaccine peptide is designed. Computational analyses indicate strong interactions and robust immune responses induced by the vaccine. While these *in silico* studies provide a promising foundation, the efficacy of the engineered vaccine candidate requires further investigation. This research represents a crucial step towards the development of a much-needed preventive measure against monkeypox.

The riboflavin biosynthetic pathway emerges as a promising frontier in the battle against resistant bacteria. Given its absence in humans, researchers are investigating the enzymes within this pathway as potential targets for drug development. In their endeavor to combat bacterial resistance, Islam and Kumar review systematically examines the structural details, active site architectures, and molecular mechanisms of catalysis involving the seven distinct enzymes. Beyond providing insights into potential drug targets, the comprehensive review surveys small molecule inhibitors developed against these enzymes. By consolidating existing research, the review serves as a valuable resource for researchers seeking to design inhibitors against resistant bacterial strains. The emphasis on structural details and catalytic mechanisms positions this review as a foundational guide for the rational development of inhibitors targeting the riboflavin biosynthetic pathway.

Nanoparticles are gaining recognition for their potential role in addressing the challenges posed by antibiotic resistance. Specifically, engineered nanoparticles like silver and selenium nanoparticles showcase unique antimicrobial properties that surpass traditional antibiotic limitations. In a study addressing *Pseudomonas aeruginosa’s* notorious drug resistance, Kanak et al. introduce selenium nanoparticles (SeNPs) as an unconventional contender. Through a computational approach, the research investigates the binding interaction pattern between SeNPs and essential quorum sensing proteins in *P. aeruginosa*. The findings suggest SeNPs play a dual role, acting as both quorum sensing inhibitors and antimicrobial agents. The ‘locked’ interaction pattern identified computationally implies a potential disruption in the quorum sensing mechanism, inhibiting acyl homoserine lactones (AHLs) production and impeding the activation of virulence factors. This study not only introduces SeNPs as a potential solution against *P. aeruginosa* infections but also underscores the significance of understanding the mechanistic interactions between nanoparticles and bacterial signaling pathways. Meanwhile, Kumar et al., in their quest for effective antimicrobial agents, explore silver nanoparticles (AA-AgNPs) synthesized from *Abroma augusta* leaf extract. This environmentally friendly synthesis method highlights the potential of AA-AgNPs as a solution against multidrug-resistant bacteria. The study meticulously characterizes AA-AgNPs, demonstrating robust antibacterial and antibiofilm activities against MRSA and VRE. With its green synthesis, comprehensive characterization, and antibacterial assessments, this study positions AA-AgNPs as promising candidates for further toxicity studies and exploration as potential medical remedies. As the field holds promise, careful consideration of potential side effects and environmental impacts is crucial as nanoparticles continue to be explored as a strategy in the fight against antibiotic-resistant bacteria.

The collective findings presented in this editorial underscore the multifaceted efforts to address the challenges posed by infectious diseases, antibiotic resistance, and the urgent need for effective vaccines. From unraveling the molecular intricacies of bacterial persistence to exploring novel antimicrobial agents and designing innovative vaccines, these research articles contribute significantly to the ongoing battle against resilient pathogens. Looking forward, these studies highlight the need for rigorous experimental validations, optimization of identified candidates, exploration of combination therapies, cross-disciplinary collaboration, global surveillance, policy implementation, and public awareness to translate these discoveries into practical interventions that safeguard public health in the face of evolving microbial challenges. The future holds promise for a holistic approach that combines cutting-edge research with practical applications to combat infectious diseases and antibiotic resistance on a global scale.

